# A novel data balancing approach and a deep fractal network with light gradient boosting approach for theft detection in smart grids

**DOI:** 10.1016/j.heliyon.2023.e18928

**Published:** 2023-08-15

**Authors:** Afrah Naeem, Nadeem Javaid, Zeeshan Aslam, Muhammad Imran Nadeem, Kanwal Ahmed, Yazeed Yasin Ghadi, Tahani Jaser Alahmadi, Nivin A. Ghamry, Sayed M. Eldin

**Affiliations:** a Department of Computer Science, Bahria University, Islamabad 44000, Pakistan; b Department of Computer Science, COMSATS University Islamabad, Islamabad 440000, Pakistan; c School of Computer and Artificial Intelligence, Zhengzhou University, Zhengzhou 450001, China; d Department of Computer Science, Al Ain University, United Arab Emirates; e Information systems department, College of Computer and Information Sciences, Princess Nourah bint Abdulrahman University, 11671, Riyadh, Saudi Arabia; f Cairo university, Faculty of Computers and Artificial Intelligence, Giza, Egypt; g Center of Research, Faculty of Engineering, Future University in Egypt, New Cairo 11835, Egypt

**Keywords:** Deep learning, ELectricity theft detection, Fractal network, Hybrid sampling, Light boosting method, Smart grids

## Abstract

Electricity theft is the largest type of non-technical losses faced by power utilities around the globe. It not only raises revenue losses to the utilities but also leads to lethal fires and electric shocks at distribution side. In the past, field operation groups were sent by the utilities to conduct inspections of suspicions electric equipments stated by the public. Advanced metering infrastructure based recent development in the smart grids makes it easy to detect electricity thefts. However, the conventional supervised learning techniques have low theft detection performance mainly due to imbalance datasets available for training. Therefore, in this paper, we develop a novel theft detection model with twofold contribution. A unique hybrid sampling technique named as hybrid oversampling and undersampling using both classes (HOUBC) is proposed to balance the dataset. HOUBC first performs undersampling and then oversampling using both the majority (normal) and minority (theft) classes. A new deep learning method, fractal network is applied with light gradient boosting method to extract and learn important characteristics from electricity consumption profiles for identifying electricity thieves. The proposed model relies on smart meter's data for theft detection and hence, a rapid and widespread adaption of this model is feasible, which shows its main advantage. The performance of the model is evaluated with real-world smart meter's data, i.e., state grid corporation of China. Comprehensive simulation results describe the effectiveness of the proposed model against conventional schemes in terms of electricity theft detection.

## 1 Introduction

Electricity theft not only endangers human lives such as risks of fire and electricity shocks but also leads to significant revenue loss. It occurs when electricity users manipulate the smart meters to reduce electricity bills and bypass connection of the smart meters. These practices raise the financial burden for both legitimate electricity users and power utilities. Nonetheless, power utility companies face two types of power losses. Technical losses (TL) and non-technical losses (NTL). The first TL occur when there is energy dissipation in the transformers and transmission lines. Whereas, NTL is caused due to billing errors, faulty meters and electricity theft [1]. Based on statistics, utilities incur revenue losses of 0.5% to 3.5% per year due to electricity theft in the United States [2].

To alleviate the revenue losses caused by electricity theft, several solutions have been proposed in the recent literature for electricity theft detection (ETD). Traditional methods rely on labor-intensive inspection, which is tedious and time-consuming task. This inspection can be replaced by advanced methods, which rely on electricity consumption (EC) data obtained from smart meters [3]. However, the enhancement of advanced metering infrastructure (AMI) in the smart grids leads to new electricity theft attacks. The stealing of energy becomes easy with the debut of AMI and therefore, it is predominant in smart grids as compared to traditional grids [4]. These electricity theft attacks can be categorized into three groups: 1) cyber-attacks that are being done within smart meters over the network; 2) physical-attacks in which consumers physically tamper their meters to reduce electricity bills and disconnect or reverse the meters to reduce the load; and 3) data-attacks that occur through cyber and physical-attacks with the aim of manipulating measurement values. All these types of attacks can be detected through analysis of the consumers' EC patterns [5].

The existing methods used for ETD are broadly categorized into hardware-based methods and data-driven methods. Hardware-based methods use sensors and micro-controller systems to detect electricity theft. Although, due to the high cost of development and maintenance, these methods cannot be adopted as efficient methods. Contrarily, the data-driven based ETD methods have acquired ample attention from researchers in the past few years [6]. These methods leverage a variety of machine learning techniques to identify the anomalous electricity consumption behavior of consumers using classifiers. However, these methods request for a large amount of data, which increases the training period of classifiers. These methods also require retraining with respect to the changes in conditions such as occurrence of new theft type [7]. Moreover, a large number of labeled theft cases required by supervised learning models are rarely exist in the real-world scenarios. Despite of the issues in data-driven methods, they are feasible to utilize in real-world and are successful in achieving wonderful performance in short period of time.

Literature is teemed with supervised and unsupervised learning techniques for NTL detection. The widely adopted supervised learning methods include convolutional neural network with long short-term memory (CNN-LSTM) [6], LSTM based boosting [8], gated recurrent unit (GRU) [9], UNet model [10], wide and deep convolutional neural network (W&D CNN) [3], auto-regressive integrated moving average [11], ensemble bagged tree [12], multiple linear regression [13], gradient boosting theft detector [14], support vector machine (SVM) [15], [16] etc., which are used in the literature for detecting electricity fraudsters. Alternatively, unsupervised methods used for ETD are entropy-based detection [17], K-means clustering-based model [18], self-organizing map (SOM) [4], fuzzy logic or clustering [19], [20], LSTM-Gaussian mixture model [21], Markov-chain model [22], autoencoders [23], etc. Moreover, there are also semi-supervised methods that use both labeled and unlabeled data to detect inspected and un-inspected theft cases [24], [25], [26], [27]. Most of these models are less accurate in terms of ETD with high computational time and dependence on the domain knowledge to perform feature selection and extraction.

In the existing literature of ETD, the common issue discussed but seldom solved is the imbalanced data problem. Two generic data sampling strategies found in the literature are oversampling and undersampling. Some of the broadly accepted methods for oversampling include synthetic minority oversampling technique (SMOTE), borderline oversampling with SVM, random oversampling, borderline-SMOTE and adaptive synthetic sampling [28]. All these methods follow the concept of alleviating imbalance ratio by synthesizing samples of the minority (theft) class. However, random generation of data replicates existing samples, which are likely to overfit the model. SMOTE generates new instances of electricity consumers to balance the data. However, newly generated instances do not belong to the actual consumption of residential consumers due to the addition of noise. On the other hand, few techniques used for undersampling include condensed nearest neighbor rule, one-sided selection, neighborhood cleansing rule, near miss and Tomek links undersampling [28]. These methods follow the convention of lessening samples in the majority (honest) class to balance the dataset. Although, they discard useful information from the majority (honest) class, which could be necessary to train a classifier. It also causes underfitting problem. Sampling methods are judgmental tasks as there exist chances of biasness due to the wrong selection of samples. This wrong selection will make the whole process ineffective. Hybrid methods such as adaptive sampling boosting and normal-distribution with similarity-based method [28], are also developed to overcome the issues of oversampling and undersampling techniques.

### 1.1 Problem analysis

By analyzing consumers' previous EC behavior, it becomes easy for the supervised learning methods to detect electricity theft. However, existing methods have low detection accuracy due to the predominant issue of imbalanced data available for training. Specifically, the number of fair consumers is remarkably higher than the electricity thieves. This problem of imbalanced data (i.e., underrepresentation of one class) is a major concern in supervised machine learning, which is the most extensively adopted methodology in the literature of NTL detection [29]. Moreover, unavailability of sufficient theft data limits the performance of the supervised learning solutions, resulting in low detection rate. Therefore, to handle the class imbalance problem in the context of NTL detection, little attention has been paid in the literature. This potentially raises the need for an efficient and a cost-effective solution to solve the above-mentioned class underrepresentation problem.

### 1.2 Contributions

Following are the contributions of the paper.

1. *Novel hybrid oversampling and undersampling using both classes (HOUBC) technique*: To overcome the limitation of class biasness, a new sampling technique HOUBC is proposed that solves the imbalanced data problem. This hybrid technique first undersamples the data from majority class, then oversamples the data using both majority and minority classes. HOUBC does not only resolve the overfitting problem by generating distinct samples but also consider resemblance with the realistic energy theft data. It also enhances learning ability of the supervised learning methods.

2. *Proposed STL-FractalNet-LightGBM model*: A new model is proposed which is a combination of seasonal and trend decomposition using loess (STL), fractal network (FractalNet) and light gradient boosting machine (LightGBM). The preprocessed data is first given as input to STL method that separates seasonality and trend from consumer's data pattern. Then, this separated data is passed to FractalNet for better generalization and memorization. LightGBM is applied for classification results and for improving the learning ability of FractalNet based on loss function.

3. *Inclusive simulations*: A number of simulations are performed with different values of parameters to find optimal values on which our proposed model and benchmark methods perform the best for ETD. The proposed model is then compared with various conventional methods to verify its effectiveness using seven performance metrics. These metrics are area under the receiver operating characteristic curve (AUC-ROC), precision-recall area under the curve (PR-AUC), precision, recall, accuracy, Matthews correlation coefficient (MCC) and F1-score.

## 2 System methodology

**Figure 1:**
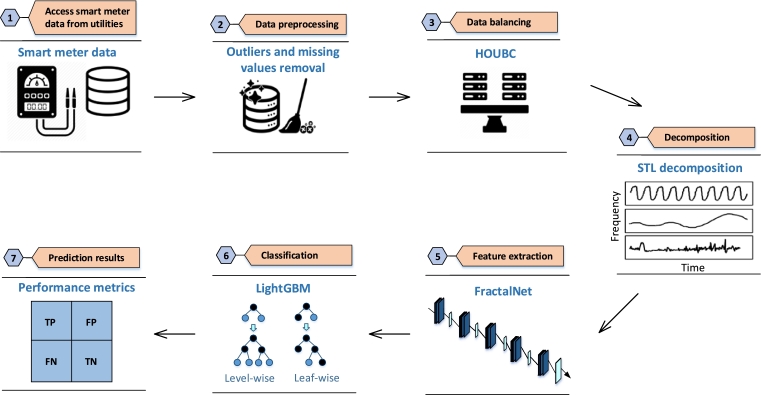
Overview of the proposed system model.

Electricity theft is one of the major threats in AMI that does not only affect revenue of the utility companies but also economy of a country. So, there is need for an efficient solution that deals with these threats and helps in providing a reliable supply of energy to the consumers. Therefore, a more secure, efficient and reliable solution is proposed for ETD in this paper. The proposed solution is composed of four main steps, as shown in Fig. 1. These steps are: 1) data preprocessing that deals with missing values, outliers and data imbalance issues before passing data to the model for learning and prediction, 2) the preprocessed data is passed to STL for decomposition, 3) the decomposed data is passed to the FractalNet for feature extraction and then LightGBM is used as final classifier, 4) suitable performance indicators are then employed to fairly assess the performance of the proposed model for ETD.

### 2.1 Data preprocessing and cleansing

To apply the proposed model for NTL detection, we first clean the raw data. Real-time energy consumption data usually contains missing values due to several reasons such as storage issues of the system, failure of smart meters, unscheduled maintenance, poor signal issues and if there are problems in sending or receiving endpoints. Two kinds of missing values are found in the dataset: missing channel data, which occurs when no information is stored in the channel and missing interval data, which is related to transmission problems [30], [31]. Consequently, the simplest solution so far is to either remove the observations containing missing values or replace these values by fill-in methods. Therefore, this paper presents a linear interpolation method to handle missing values using the formula as given in equation (1) [3]:

(1) $$f(x_{i,t})=\left\{ \begin{array}{c} \frac{x_{i,t-1}+x_{i,t+1}}{2}\;x_{i,t}\in NaN,x_{i,t-1},x_{i,t+1}\notin NaN,\\ 0\;x_{i,t}\in NaN,x_{i,t-1}\;\text{or}\;x_{i,t+1}\in NaN,\\ x_{i,t}\;x_{i,t}\notin NaN, \end{array}\right.$$

where, $x_{i,t}$ is the value of consumption data that is represented as *NaN* in the case of null or non-numeric character.

Z-score-based method such as “Three-sigma rule of thumb” [3] is an effective method to deal with the outliers. However, it is convenient only for small size datasets. So, we choose isolation forest method (IFM) [32] to deal with the outliers after recovering missing values. This method is based on decision trees. To build a single tree, IFM picks one feature from the feature space and performs its random splitting. Values ranging between minimum and maximum is called path length. This step is performed for all training data. All trees are then ensembled to create a forest. To make a prediction, IFM takes one observation and compares it with a random splitting value in a node. Such node has two children nodes where further comparisons can be made. Each observation is assigned a score between 0 and 1, where 0 means that the observation is normal and 1 means that the observation is different. This method has few parameters that make it fairly robust and easy to optimize. After dealing with the outliers, data is normalized to make sure that each feature in the feature space lies on the same scale. Data normalization is necessary as neural networks are sensitive to diverse data. Therefore, we apply min-max normalization method [4] to standardize the data.

Data sampling is one of the common methods to deal with the data imbalance issue in supervised machine learning methods. After data normalization, a proposed sampling method HOUBC is applied in this study. This method is based on the concept of random oversampling and random undersampling method as this technique also selects random users' consumption from the dataset. However, the logic behind the creating or discarding the samples after choosing these random samples is different in our case. The pseudocode of this method is given in Algorithm 1, in which input variables are given as: dataset *S*, minority class *y* with consumers labeled as 1 and majority class *z* consumers labeled as 0. Whereas, minority class and majority class are interchangeably represented as theft class and honest class, respectively.

**Algorithm 1:**
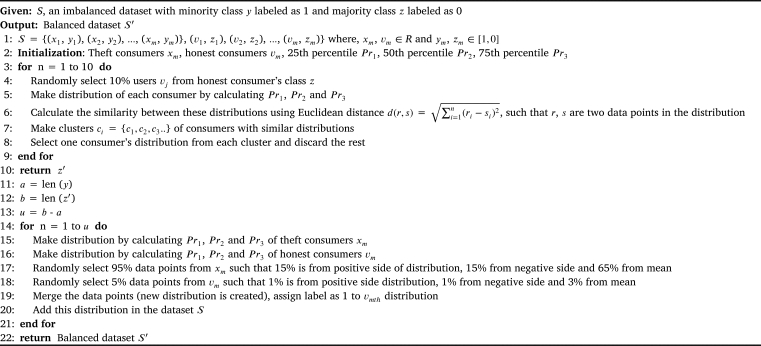
HOUBC technique for class balancing in ETD.

HOUBC has two parts: undersampling and oversampling. Firstly in undersampling, we randomly select 10% of consumers' consumption from data of the honest consumers' class *z*. Then, the data distribution of each consumer is created using percentiles. Similarity index of two consumers' distribution pattern is then calculated using Euclidean distance formula [33]. This index is measured to check the similarities in the consumer's consumption patterns. The number of consumers whose EC patterns have more resemblance is put into the same cluster. After clustering, one consumer is selected from each cluster as all the consumers possess same distribution pattern that causes overfitting. Whereas, rest of the consumers in the clusters are discarded. This process is performed for 10 iterations. Now the updated majority class becomes $z^{\prime }$. After completion of the undersampling process, oversampling is performed.

The total number of consumers (cases) is calculated from the dataset *S* for example, it contains 1000 consumers. Thereafter, the numbers of both honest *y* and theft consumers $z^{\prime }$ are counted and assigned to temporary variables a and b interchangeably. The difference between them is stored in *u*, i.e., 980 (honest) - 20 (theft) = 960 samples that need to be created for balancing the data. The consumption distributions of both minority class *y* and majority class $z^{\prime }$ are created and divided using percentiles $Pr_{1}$, $Pr_{2}$ and $Pr_{3}$. Then, percentage of the data residing in the percentiles of both distributions is calculated. We then randomly select 5% and 95% data points from both distributions, i.e., honest and theft consumers' data, respectively. After selection, we merge the selected data points and make a new distribution. The newly created samples are added to the dataset. The process is repeated until *y* becomes equal to $z^{\prime }$. This mechanism of oversampling shows how theft samples are created. So, by doing sampling of the data in this way, it overcomes the previous limitations of divergence from the actual data, overfitting in oversampling and insufficient learning when undersampling of data is done. We collect 95% (15+15+65) data points from the theft class and only 5% (1+1+3) points from honest class for oversampling. The reason is that choosing data points from the theft class leads to overfitting and synthetic generation of samples will diverge the data from actual data. The proposed model will be better able to learn the normal and abnormal consumption.

**Figure 2:**
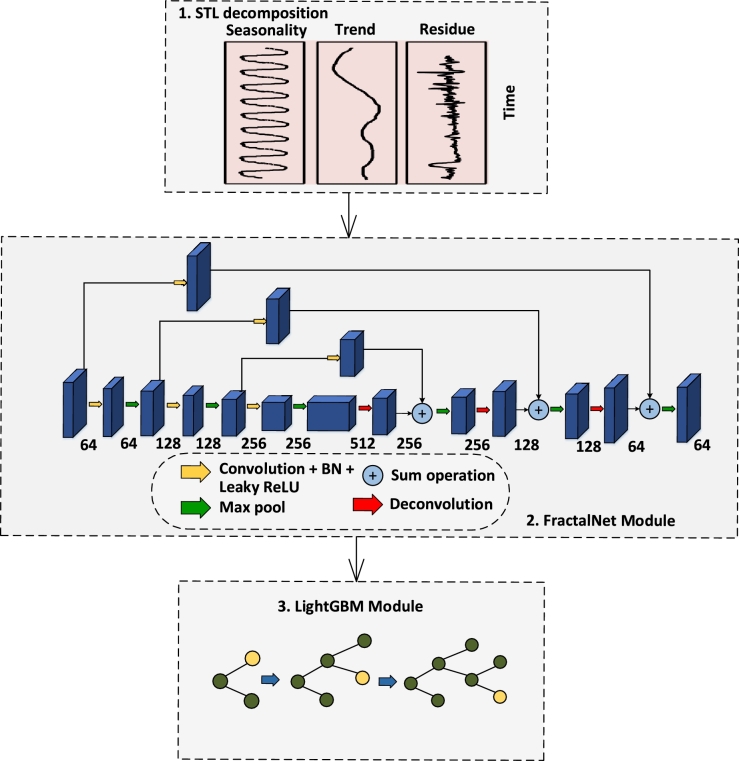
Proposed STL-FractalNet-LightGBM model.

Fig. 2 presents a more detailed illustration of the proposed model. STL method is applied for decomposing time series data into seasonality and trend, so that FractalNet model will be better able to learn seasonality and trend of both honest and theft consumers' profiles. After decomposition, FractalNet is applied for refined feature extraction. LightGBM is then applied for classification and to improve the performance of FractalNet by learning from the previous mistakes.

### 2.2 FractalNet module

**Figure 3:**
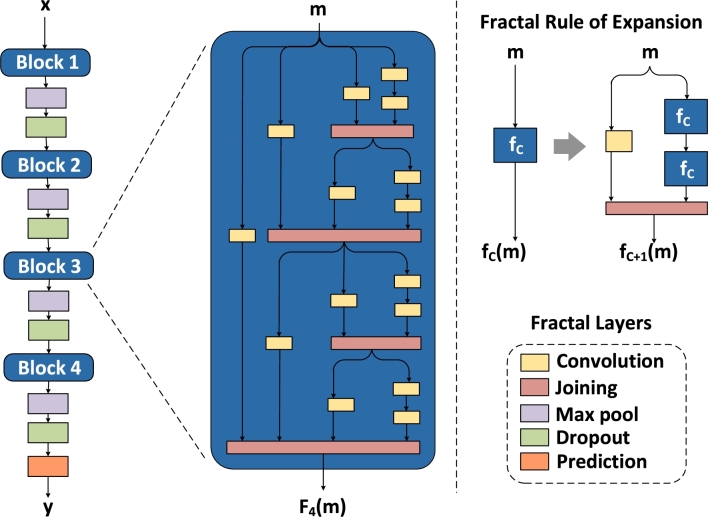
Detailed FractalNet model architecture.

Fig. 3 shows the building block of the FractalNet architecture. This model is built upon the idea of using non-residual deep network and drop-paths to reduce overfitting and regularize the co-adaption of sub paths in the FractalNet structure [34]. It has an interesting property that with shallow subnetworks, it performs efficiently and by increasing depth of the subnetworks, it yields more accurate results.

For the ground case, $f_{1}(m)$ is the single convolutional layer which is calculated by $f_{1}(m)$ = conv(m). Then recursive fractals $f_{C+1}(m)$ are calculated using equation (2) [34]:

(2) $$f_{C+1}(m)=[(f_{C}◯f_{C})(m)]⊕[conv(m)]$$

where, *C* is the number of columns of the truncated fractal $f_{C}$, ◯ denotes composition and ⊕ represents the joint operation that merges output features of two convolutional layers into one. The total number of convolutional layers of the deepest path within a block is $2^{\left(C-1\right)}$, where *C* = 4, which means that there are total 8 layers. For the joining layer (pink), element-wise mean is computed instead of concatenation or addition. At the left side of Fig. 3, FractalNet is cascaded with four blocks (b = 4). Then the total number of convolutional layers in the deepest path of the whole network is b × $2^{\left(C-1\right)}$, which means that there are 32 layers in the entire network. Between the two blocks, 2 × 2 max pooling layer and dropout layer are used to reduce both the size of feature maps and dependency of the network on specific neurons. Batch normalization and leaky ReLU are used after each convolution. Drop paths (as regularization strategy) are of two types: local and global drop paths. In global drop paths, one path is selected for the entire network. On the other hand, local drop paths have fixed probabilities to drop the inputs. It ensures that at least one path will survive. Drop-paths prevent co-adaption of parallel paths in a block by randomly dropping operands of the joining layer. Lastly, LightGBM uses leaf-wise generation strategy that can reduce training loss when growing the same leaf [35]. We use it as a classifier for final results as well as for improving the learning ability of the weak learners on the basis of training loss calculated. So, it gives 0 as output for fair consumption and 1 if theft is detected.

## 3 Simulation results and discussion

### 3.1 Experimentation setup

The Python scripts used for the proposed scheme are as follows:

1. Scikit−learn library is used for outlier detection and normalization. IFM is applied for removing outliers from the data. Furthermore, after removing the erroneous values, min-max normalization is applied to scale the data.

2. After preliminary processing, theft profiles are generated using the HOUBC strategy. FractalNet is built and trained using an open source library, i.e., TensorFlow [36].

### 3.2 Dataset availability

All the simulations are performed on the real smart meters' data released by state grid corporation of China (SGCC)^1^ and available on cite^2^ [3]. Specifically, this dataset contains the record of 42,372 electricity consumers within the period of three years (from January 1, 2014 to October 31, 2016). The dataset is sorted according to dates. Column represents features in the form of dates, which means that it is a multi-variate dataset. Whereas, the EC values are observations given in the rows. Although, it contains some missing and erroneous values that need to be handled. It is a highly imbalanced data as it contains 38,757 honest consumers and only 3,615 dishonest consumers occupying nearly 9% of all the consumers. This implies that the electricity theft rate is quite serious in China.

### 3.3 Evaluation criteria

One of the most difficult challenges to tackle electricity theft is to choose suitable metrics for the evaluation of supervised machine learning models as the data is usually imbalanced. Attention should be paid in considering a metric that is suitable for this type of data. AUC is the most widely used performance metric to evaluate classification accuracy of a binary classifier at various threshold settings. It measures the quality of the model's separability, i.e., how good it is at distinguishing between classes. An average classifier has 0.5 score of AUC. Whereas, an efficient classifier presents the AUC score near to 1. It is calculated by using the formula as given in [29]. ROC is the probability curve with two parameters, i.e., true positive rate (TPR) and false positive rate (FPR). MCC [29] is also used for evaluating the performance of binary classifiers. It takes into account all the four outcomes of a confusion matrix, which indicate a reliable assessment of a classifier with imbalanced data. The values of MCC ranges between -1 to 1, i.e., from perfect prediction to completely inaccurate prediction. The objective of ETD detection is to increase TPR while, decreasing the FPR.

### 3.4 Simulation settings of the proposed model

All neural network models strongly rely on hyper-parameters, so we fine-tune their values and control the size of filters and hidden layers. We set the values using grid-search approach and monitor the performance of the proposed solution using the validation dataset. Table 1 shows the range of parameter values for our proposed model. Dropout and pooling layers are used after every fractal block. We set FractalNet with four blocks. Moreover, 2 × 2 non-overlapping max-pooling layer, dropout layer and subsampling are applied after each block. This reduces spatial resolution over the duration of the entire network. The number of layers selected for the FractalNet model is 32. By increasing layers to 60 or above, the number of parameters increases and the demand for large size data also increases, which further results in overfitting, high computational time and high error rate.

**Table 1:** Hyperparameter settings of proposed model.

FractalNet Module		

Parameters	Values range	Optimal value

N (layers)	20, 32, 60, 80	32
Kernel size	3, 4	3
Activation function	Sigmoid, ReLU, Leaky ReLU	Leaky ReLU
Dropout	0.01, 0.1	0.01
Pooling	Average, max	max
Strides	2, 3	2

LightGBM Module		

Parameters	Values range	Optimal value

max_depth	5, 10	5
lambda_l1	0.1	0.1
lambda_l2	0.001, 0.01	0.01
learning_rate	0.01, 0.1	0.1

Adam Optimizer		

Parameters	Values range	Optimal value

learning_rate	0.001, 0.01	0.001

For LightGBM, three parameters are tuned. Maximum depth set for this method is 5, which limits the depth of the tree model. It is used to deal with overfitting problem for small sized datasets. Alpha is the learning rate, which means higher value of alpha results in faster initial training. Adam optimizer is used as it can handle sparse gradient on noisy data problems. It is a robust optimization technique, computationally less expensive and requires little memory. The configuration of Adam parameters is shown in Table 1.

### 3.5 FractalNet model results

**Figure 4:**
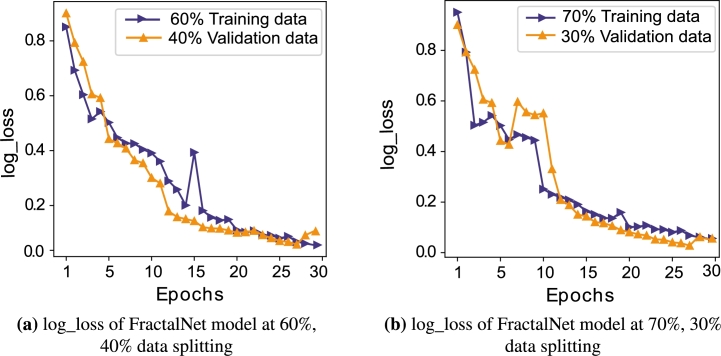
log_loss of FractalNet model on different data splitting ratios.

To assess the performance of the proposed model, extensive experiments are performed. Fig. 4 shows the performance of our proposed model in terms of loss function. Here, two different splitting of dataset are considered, where proposed model is trained alternatively. SGCC dataset is first split into 60% training data and 40% validation data as shown in Fig. 4(a) to evaluate the model's performance. The number of epochs is set to 30 to show clear representation of loss function values of the complex model at each epoch. As the number of epochs increases, the logarithmic loss (log_loss) decreases gradually from 0.85 to 0.15 on both training and validation data.

However, there is a slight increase in loss of 0.4 at $15^{th}$ epoch during training due to overfitting, except this, the curve is visualized as a smooth curve. The difference between training and validation loss is minimum, which means that our model achieves best results for unseen data.

The performance of the proposed model is also assessed by dividing the dataset into 70% training data and 30% validation data as shown in Fig. 4(b). While, using 70% training data, training loss of the model ranges from 0.84 to 0.07. Although, there is an abrupt change in loss function during epochs 3 to 12 on both training and validation data. The curve becomes smooth after epoch 15, which dictates well learning adaptability of the model. At $30^{th}$ epoch, log_loss of the FractalNet method on training set is same as the loss on validation set. It can be seen that irrespective of increasing the ratio of training of the model, it performs better on validation data in both cases and continue to minimize loss at later epochs.

**Figure 5:**
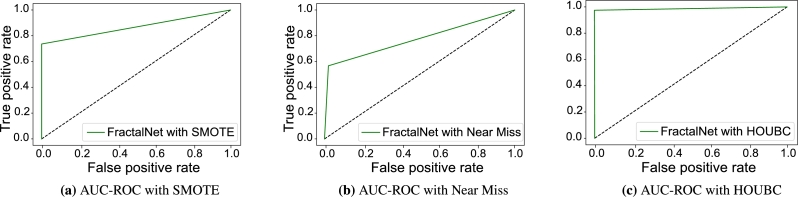
AUC-ROC curve of FractalNet model with SMOTE and HOUBC.

**Figure 6:**
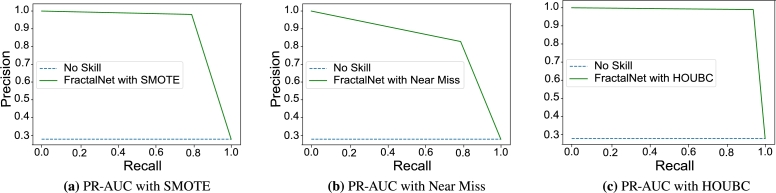
PR-AUC curve of proposed FractalNet model with SMOTE and HOUBC.

Furthermore, AUC-ROC and PR-AUC curves of the proposed model are also examined with existing SMOTE and Near Miss sampling technique. AUC-ROC score with SMOTE method is 0.761 and with Near Miss undersampling AUC-ROC is 0.58 which is quite low as shown in Fig. 5(a) and Fig. 5(b). Similarly, AUC-ROC is 0.921 when applying HOUBC method for sampling as displayed in Fig. 5(c). PR-AUC without HOUBC technique is shown in Fig. 6(a) and Fig. 6(b) whose value is 0.77 and 0.67 for the proposed model. On the other side, PR-AUC gives the score of 0.904 with HOUBC on validation dataset, which means that the proposed model gives outstanding results with HOUBC sampling technique as displayed in Fig. 6(c).

The proposed model is employed for the enhanced performance in terms of electricity theft detection thereby not majorly focusing on the computational cost. The computational cost of FractalNet is reduced by using it with light boosting method to learn from mistakes and give accurate results. It also has reduced the burden of manual feature extraction as FractalNet have the capability to extract the features with less computational cost. Although, FractalNet being deep learning model has computational time complexity of 1.5 h because of hardware constraints i.e., unavailability of graphical processing unit. The main aim of ETD is to perform accurate prediction i.e., false positive rate rather than computational complexity of the models.

### 3.6 Simulation settings for benchmark methods

The performance of the proposed model is compared with benchmark models for ETD. The benchmarks used for comparison are DenseNet, W&D CNN [3], CNN-LSTM [6], GRU [9], hybrid LSTM [23], XGBoost and SVM. We set generic parameters of the traditional models and proposed model at the values where it performs best at training. Extensive simulations are performed to find the appropriate values of hyperparameters on which proposed model performs the best. Specifically, we do not employ any mechanism to tune the hyperparameters of the proposed solution because it is a deep neural network, which requires extensive time to find optimal hyperparameters' values. Therefore, the proposed solution is trained and tested on its best hyperparameters' configuration where it efficiently minimizes the loss and improves the ETD results using real EC data. The same strategy is applied to the benchmark methods. The hyperparameter settings of the benchmarks are given in Table 2.

**Table 2:** Hyperparameters of benchmark methods.

Hyperparameters setting		

Methods	Input data	Parameters setting

XGBoost	1-D	Learning rate: 0.01, Maximum depth: 9, Number of trees: 1000, Min child wgt: 10

SVM	1-D	C: 1.0, gamma: 0.1

CNN-LSTM	1-D, 2-D	N (layers): 10, Number of units: 30, Leaky ReLU-alpha: 0.001, Dropout: 0.1

LSTM-MLP	1-D	N (layers): 4, Number of units: 256, LSTM layers: 512, MLP layers: 512, Dropout: 0.1

DenseNet-FCN	2-D	N (layers): 67, Number of filters: 64, Kernel size: 3, Activation function: Leaky ReLU, Dropout: 0.1, Pooling: max, Strides: 2

GRU	1-D	N (layers): 10, Number of units: 30, Leaky ReLU-alpha: 0.001, Dropout: 0.1

### 3.7 Comparison and discussion

**Figure 7:**
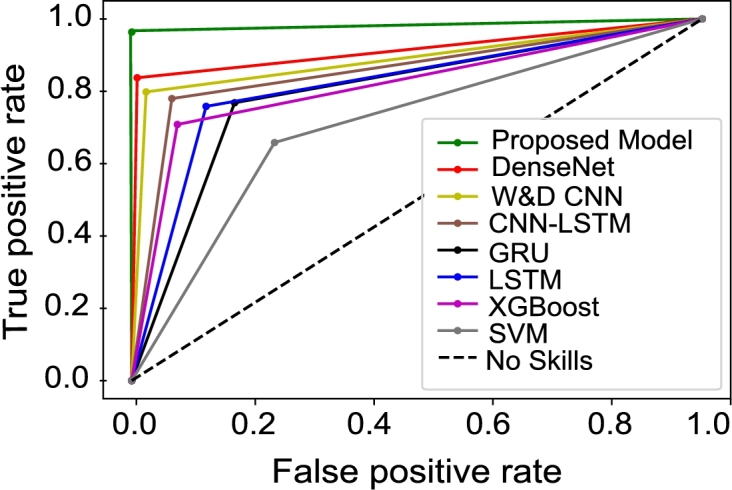
Comparison between TPR and FPR for all models.

**Figure 8:**
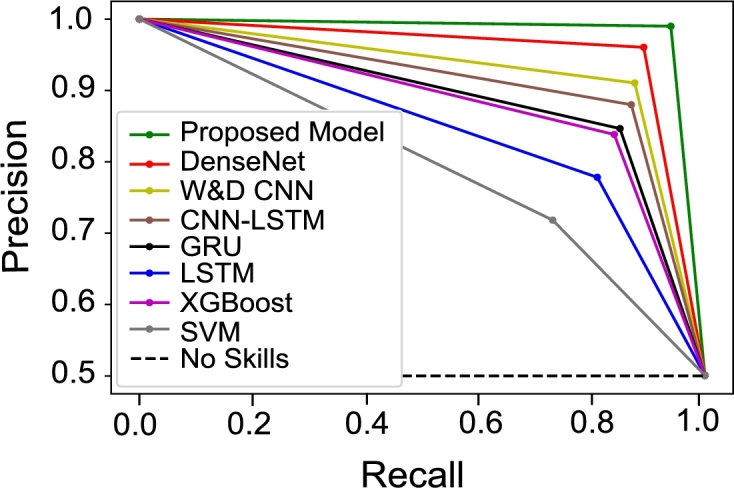
Results for PR-AUC curve comparison of all models.

**Figure 9:**
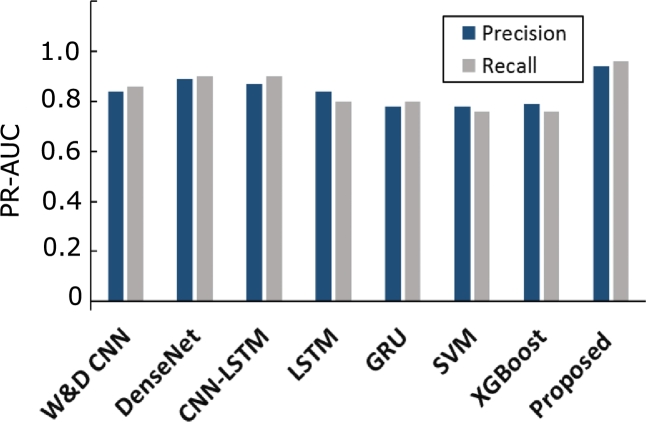
Comparison between precision and recall based on PR-AUC for all models.

In this section, we compare the proposed model with widely adopted and most recent ETD methods mentioned in the literature. For a fair comparison, the proposed preprocessing steps are applied to all models and the performances of the models are evaluated using SGCC dataset. AUC-ROC score of the proposed model is 0.921 as shown in Fig. 7. Whereas, DenseNet, W&D CNN and CNN-LSTM have AUC-ROC score ranges between 0.78-0.82 with less FPR. However, other models such as XGBoost, GRU and LSTM have a high FPR with AUC-ROC score of 0.65-85. The reason is that hybrid models show improved performance as compared to single classification models. The probability curve of our proposed model covers more area by learning from past mistakes and gives better results. It is also observed in Fig. 8 that the PR-AUC value of our proposed model is approximately 1, as compared to other models. This means that the proposed model has the capability to accurately detect fair consumers despite of the unusual changes in EC. Moreover, other models have PR-AUC scores ranging between 0.7-0.85, except the SVM model, whose performance is worst in terms of precision and recall. It can be seen from AUC-ROC and PR-AUC results that our model performs the best by accurately detecting electricity theft and honest cases. Bar chart in Fig. 9 briefly describes that the SVM does not perform well on the imbalanced dataset. Even though, balanced data is given to improve its performance ability; however, it still gives underrated performance because of high dimensional data. Table 3 shows that the proposed model achieves 0.921, 0.942, 0.933, 0.942, 0.961, 0.904 and 0.962 for AUC-ROC score, MCC score, F1-score, precision, recall, PR-AUC score and accuracy, respectively. Besides this, other existing models perform well in terms of precision whose values range between 0.70-0.85. Nonetheless, our proposed model outperforms all these models.

**Table 3:** Comparison of the proposed model with benchmarks based on seven performance metrics.

Metrics	DenseNet	W&D CNN	CNN-LSTM	GRU	Hybrid-LSTM	XGBoost	SVM	Proposed Model
AUC-ROC	0.841	0.802	0.906	0.748	0.866	0.752	0.658	0.921
MCC	0.902	0.803	0.817	0.748	0.732	0.756	0.678	0.942
F1-score	0.924	0.823	0.901	0.784	0.869	0.762	0.648	0.933
Precision	0.892	0.84	0.870	0.788	0.849	0.793	0.688	0.942
Recall	0.903	0.861	0.905	0.808	0.826	0.752	0.667	0.961
PR-AUC	0.844	0.846	0.853	0.908	0.812	0.756	0.652	0.904
Accuracy	0.958	0.865	0.906	0.848	0.866	0.804	0.783	0.962

## 4 Conclusion and future work

This paper presents a unique sampling technique, HOUBC to sample the data so that both honest and electricity theft cases are equally learned by supervised machine learning methods. This paper also demonstrates the usage of a new deep learning model, FractalNet with LightGBM for ETD. In general, the proposed model mainly consists of STL method that separates seasonality and trend in customer EC patterns. FractalNet module performs further feature extraction and learns those features. Finally, LightGBM is used to give final classification results and to boost weak learners. We conduct comprehensive simulations on realistic smart meters' data provided by SGCC. The simulations show that the proposed model outperforms existing methods such as W&D CNN, DenseNet, CNN-LSTM, hybrid LSTM, GRU, XGBoost and SVM in terms of low FPR and high TPR. Our proposed solution can be generally applied to other scenarios, especially for industrial or economic applications. For future work, we plan to reduce computational complexity of the proposed model and then compare its computational complexity with the benchmark methods. We also plan to detect electricity theft using datasets from different countries by the proposed scheme to determine its resilience and efficacy in the electricity distribution system.

## Additional information

No additional information is available for this paper.

## CRediT authorship contribution statement

All authors listed have significantly contributed to the investigation, development and writing of this article.

## Declaration of Competing Interest

The authors declare that they have no known competing financial interests or personal relationships that could have appeared to influence the work reported in this paper.

## Data Availability

Data included in article/supplementary material/referenced in this article.

## Footnotes

1 State Grid Corporation of China http://www.sgcc.com.cn/.

2 https://github.com/henryRDlab/ElectricityTheftDetection.
